# Local activation time sampling density for atrial tachycardia contact mapping: how much is enough?

**DOI:** 10.1093/europace/eux037

**Published:** 2017-04-03

**Authors:** Steven E Williams, James L Harrison, Henry Chubb, John Whitaker, Radek Kiedrowicz, Christopher A Rinaldi, Michael Cooklin, Matthew Wright, Steven Niederer, Mark D O'Neill

**Affiliations:** 1 Division of Imaging Sciences and Biomedical Engineering, King's College London, 4th Floor North Wing, St Thomas' Hospital, 249 Westminster Bridge Road, SE1 7EH London; 2 Department of Cardiology, Guy's and St Thomas' NHS Foundation Trust, St Thomas' Hospital, 249 Westminster Bridge Road, SE1 7EH London, UK

**Keywords:** Electroanatomic mapping systems, Atrial arrhythmias, Sampling density, Local activation time mapping

## Abstract

**Aims:**

Local activation time (LAT) mapping forms the cornerstone of atrial tachycardia diagnosis. Although anatomic and positional accuracy of electroanatomic mapping (EAM) systems have been validated, the effect of electrode sampling density on LAT map reconstruction is not known. Here, we study the effect of chamber geometry and activation complexity on optimal LAT sampling density using a combined *in silico* and *in vivo* approach.

**Methods and results:**

*In vivo* 21 atrial tachycardia maps were studied in three groups: (1) focal activation, (2) macro-re-entry, and (3) localized re-entry. *In silico* activation was simulated on a 4×4cm atrial monolayer, sampled randomly at 0.25–10 points/cm^2^ and used to re-interpolate LAT maps. Activation patterns were studied in the geometrically simple porcine right atrium (RA) and complex human left atrium (LA). Activation complexity was introduced into the porcine RA by incomplete inter-caval linear ablation. In all cases, optimal sampling density was defined as the highest density resulting in minimal further error reduction in the re-interpolated maps. Optimal sampling densities for LA tachycardias were 0.67 ± 0.17 points/cm^2^ (focal activation), 1.05 ± 0.32 points/cm^2^ (macro-re-entry) and 1.23 ± 0.26 points/cm^2^ (localized re-entry), *P* = 0.0031. Increasing activation complexity was associated with increased optimal sampling density both *in silico* (focal activation 1.09 ± 0.14 points/cm^2^; re-entry 1.44 ± 0.49 points/cm^2^; spiral-wave 1.50 ± 0.34 points/cm^2^, *P* < 0.0001) and *in vivo* (porcine RA pre-ablation 0.45 ± 0.13 vs. post-ablation 0.78 ± 0.17 points/cm^2^, *P* = 0.0008). Increasing chamber geometry was also associated with increased optimal sampling density (0.61 ± 0.22 points/cm^2^ vs. 1.0 ± 0.34 points/cm^2^, *P* = 0.0015).

**Conclusion:**

Optimal sampling densities can be identified to maximize diagnostic yield of LAT maps. Greater sampling density is required to correctly reveal complex activation and represent activation across complex geometries. Overall, the optimal sampling density for LAT map interpolation defined in this study was ∼1.0–1.5 points/cm^2^.

What’s new

- Electroanatomic mapping advances allow many hundreds, or possibly thousands, of activation time points to be collected within a single local activation time (LAT) map;
- Whether such an exponential increase in LAT sampling density results in similar increases in LAT map accuracy is not known;
- This manuscript defines the optimal sampling density for atrial tachyarrhythmias as the maximal density beyond which further sampling produces minimal further improvement in interpolated LAT map accuracy;
- Optimal sampling densities are thereby determined for a variety of tachycardia mechanisms, and are found to be in the range of 1.0–1.5 points/cm^2^;
- Therefore, we propose that when a diagnosis is not reached at this density, further sampling may be less likely to improve map accuracy than a change of mapping strategy (e.g. a different window of interest, reference electrogram or LAT assignment technique).

## Introduction

Local activation time (LAT) mapping forms the of cornerstone complex atrial tachycardia diagnosis. The creation of LAT maps by electroanatomic mapping (EAM) systems involves four key steps: (1) creation of a three dimensional anatomy model, (2) analysis of intracardiac electrograms to identify activation times relative to a fixed temporal reference, (3) interpolation of the activation times across the anatomical model, and (4) representation of the interpolated data, usually in the form of a colour map. Anatomic accuracy of LA EAM chamber models has been compared to cross sectional imaging with CT/MRI,^1^ showing faithful representation of pulmonary vein dimensions and geometry. Similarly, catheter localization accuracy within EAM systems has been shown to be both reproducible and accurate.^2^ To date however, no studies have examined the accuracy of interpolated LAT maps generated with contact mapping. Rather, recommendations on the number of sampling locations required to create an accurate map are primarily based on operator experience.^3^ Meanwhile, device manufacturers continue to innovate to increase the achievable resolution of mapping systems^4^^,^^5^ but the end goal of effective diagnosis and treatment whilst minimizing procedure times remains unchanged.

Therefore, this study presents an optimal point sampling strategy for atrial tachycardias, based on systematic analysis of *in silico* and *in vivo* LAT mapping data. We defined optimal density as the sampling density required for interpolation of an accurate LAT map whilst not sacrificing efficiency by oversampling and hypothesized that an optimal activation sampling density exists beyond which further sampling is of limited clinical benefit. The impact of chamber geometry and activation pattern complexity on the optimal LAT sampling density required for mapping ATs is examined.

## Methods

### *In vivo* procedures

A total of 37 electroanatomic maps were included in the analysis. Isochronal local activation time maps were created using the Carto3 mapping system (Biosense Webster, Diamond Bar, CA, USA) by taking the activation time at each acquired point as the time from reference activation (typically an atrial coronary sinus electrogram) to the earliest rapid deflection on the bipolar mapping catheter signal.

Mapping data was analysed from 21 patients undergoing clinically-indicated ablation for left atrial tachycardia. Cases with high quality maps (defined as maps with complete LA coverage) were identified retrospectively and all identified cases were included in the analysis. Following trans-septal puncture, a 20-pole circular mapping catheter (Lasso Nav, Biosense Webster, Diamond Bar, CA, USA) was used to create a 3D geometry of the left atrium. High-density local activation time maps were constructed with the multipolar mapping catheter during tachycardia. Local activation times were automatically assigned and all points were re-annotated offline to ensure correct identification of the first component of each electrogram. Correct tachycardia diagnosis was confirmed by LAT map appearance, response to entrainment manoeuvres and response to ablation. Informed written consent was obtained from all patients for invasive procedures.

Animal studies complied fully with Danish law on animal experiments. Eight Göttingen mini-pigs (41.2 ± 7.2 kg) were pre-sedated with intramuscular azaperone (4 mg/kg) and midazolam (0.5 mg/kg). General anaesthesia was induced with intravenous ketamine (5 mg/kg) and midazolam (0.5 mg/kg). Anaesthesia was maintained with a continuous intravenous infusion of propofol (3 mg/kg/h) and fentanyl (15 μg/kg/h). Two 8F sheaths were placed percutaneously in the right femoral vein, followed by an intravenous injection of 100 IU/kg heparin. Fluoroscopy was used to position a 6F decapolar reference catheter in the coronary sinus (CS) and a 7.5F ablation catheter was advanced to the RA and used to create a LAT map during proximal CS pacing. Incomplete inter-caval linear ablation (confirmed with pace mapping) was performed, with a deliberate 5 mm gap left between cranial and caudal aspects of the lesion, before repeat LAT mapping of the new activation detour was performed.

### Atrial monolayer model

A 4 × 4cm atrial tissue model was meshed using triangular elements (84 346 triangles, 42 511 nodes, average edge length Δl = 0.2 mm). To simulate electrical activation of atrial tissue by an extracellular stimulus (1000 mV, 1 ms duration), the period of electrical activation was modelled using the bi-domain equations given by:

$$\nabla \cdot \left(\sigma_{i}\nabla \phi_{i}\right)=\;\beta I_{m}$$

$$\nabla \cdot \left(\sigma_{e}\nabla \phi_{e}\right)=\;-\beta I_{m}$$

$$I_{m}=C_{m}\frac{\partial V_{m}}{\partial t}+I_{ion}$$

$$V_{m}=\phi_{i}-\phi_{e}$$

where $\sigma_{i}$ and $\sigma_{e}$are the intra and extracellular conductance tensors, respectively, β is the surface to volume ratio (1400 cm^−^^1^), $\phi_{i}$ and $\phi_{e}$ are intra and extra cellular potentials, respectively, $I_{m}$ is the transmembrane current, $C_{m}$ is the membrane capacitance (0.01 µF mm^−^^2^), $V_{m}$ is the transmembrane potential and $I_{ion}$ is the cellular ionic current simulated using the Nygren *et al.*, atrial electrophysiology cellular model.^6^ The conduction tensors are aligned with the local tissue microstructure orientation with intracellular fibre and cross-fibre conduction set to 0.28 and 0.026 S/m, respectively. The extracellular fibre and cross-fibre conduction were set to 0.22 and 0.13 S/m, respectively, for linear and focal activation patterns.^7^ In the tissue model the fibre direction was aligned parallel to the *x*-axis. Isotropic conduction with equal intra and extracellular conductivities of 0.15 S/m was employed for re-entrant and spiral wave activation patterns in order to facilitate the production of stable re-entrant waves within the 4×4 cm monolayer domain.^8^ Following stimulation to reduce computational load, the model reverts to the mono-domain equations:

$$\nabla \cdot (\sigma \nabla V_{m})+I_{stim}=\;\beta \left(C_{m}\frac{\partial V_{m}}{\partial t}+I_{ion}\right),$$

where σ is the conductivity tensor and the fibre and cross fibre conductivity is set to the harmonic mean intra and extra cellular conductivities giving fibre and cross fibre values of 0.1232 and 0.0217 S/m, respectively (or 0.075 S/m for isotropic conduction). Electrical propagation was determined by solving the bidomain and monodomain equations using the finite element method. The model was discretized in space with linear finite elements; the non-linear term describing the ionic current was treated with a splitting technique.^9^ The ionic model was discretized in time with a forward-Euler scheme and a time step of 5 µs, while the partial differential equations were solved with a time step of d*t* = 10 µs. The numerical simulations were performed with the Cardiac Arrhythmia Research Package (CARP) solver.

To create uniform linear activation, a 2 mm region at the left edge of the tissue was stimulated. To create focal activation a 1×1mm region close to the centre of the monolayer was stimulated. To create re-entry, a circular non-conducting region (diameter 1.5 cm) was introduced in the monolayer and a dual stimulus protocol was used to initiate re-entry. To create spiral wave activation, two linear stimuli were used. The first stimulus activated the entire extent of the leftmost edge of the monolayer, whist the second critically timed stimulus initiated spiral wave re-entry. *In silico* isochronal maps (see leftmost column, *Figure **6*) were constructed from the transmembrane potential fields by calculating the time elapsed between a fixed temporal reference and the time at which the transmembrane potential exceeded a pre-defined voltage threshold (−20 mV).

**Figure 1:**
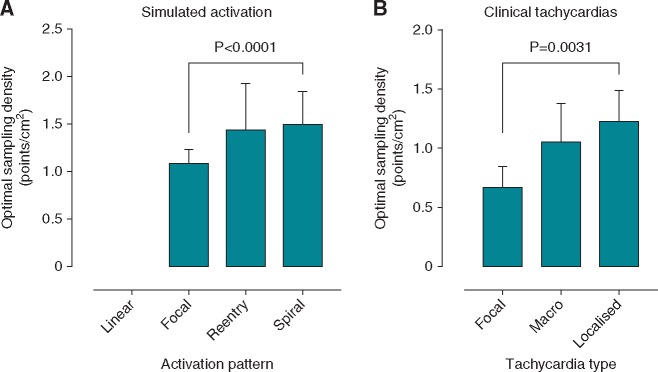
Optimal LAT sampling densities. Calculated optimal LAT sampling densities are shown for simulated activation patterns (*A*) and *in vivo* LA activation patterns (*B*). FO, focal origin; MA, macro re-entry; MI, micro (localised) re-entry.

### Local activation map construction

Re-interpolated LAT maps were created by down-sampling the number of acquired points and performing linear interpolation of the resulting activation field across the 2D domain for the simulated activation patterns or 3D geometry (i.e. electroanatomic shell) for the clinical arrhythmias. For locations beyond the outermost re-sampled points linear extrapolation based on boundary gradients was used to calculate activation times.^9^ The scatteredInterpolant class (MATLAB 8.2, The Mathworks Inc., Natick, MA) was used to implement interpolation and extrapolation methods. When performing re-interpolation on the clinical datasets, a ‘region of electrical interest’ was defined as all the nodes of the electroanatomic map lying within 10 mm of an original electrical mapping point. During re-interpolation, the interpolant was only interrogated for mesh nodes lying within this region. Points out-with this region were assigned indeterminate local activation times and are represented as grey in the accompanying figures.

### Optimal LAT sampling density measurement

For simulated activation patterns, we defined the reference map as the high-resolution isochronal map created from the activation times calculated at every mesh node. For clinical activation patterns, we defined the reference map as the LAT map created by the clinical mapping system using all available LAT points. Reference and re-interpolated maps were compared quantitatively by computing the sum of squared differences between activation times at every mesh node in the region of electrical interest of the chamber model. Results are presented per node (average LAT map error, ms/node) in order to normalize for inter-case differences in the number of nodes per geometry.

**Figure 2:**
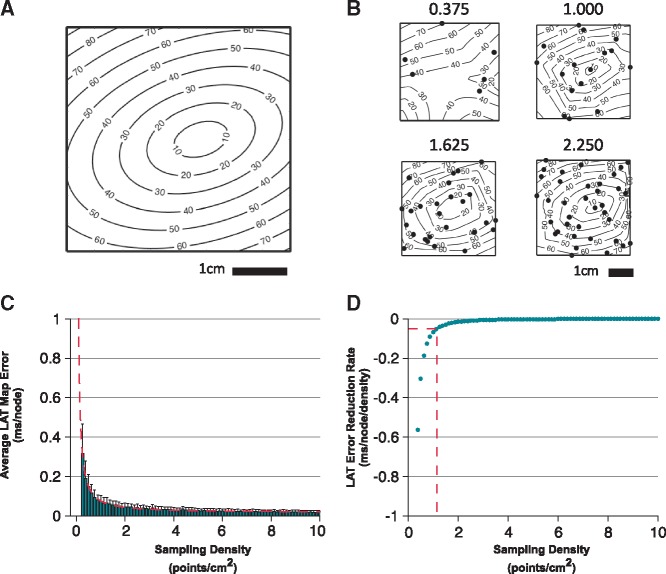
The process used for determination of optimal LAT sampling density. (*A*) The process is shown for a focal tachycardia in a 4×4 cm monolayer with fiber orientation of 18° to the *x*-axis. (*B*) Activation times are re-sampled at increasing sampling density and regional activation is re-interpolated to give the resulting ‘down-sampled’ activation maps. Sampling densities (points/cm^2^) are given above each example map. (*C*) Mean normalized LAT map error at each sampling density is calculated by comparing activation times at each node with the original activation times in A. (*D*) Taking the first-derivative of the curve in ‘*C*’ indicates the sampling density at which minimal further improvement in map accuracy is generated.

The optimal sampling density for each LAT map was defined as the highest sampling density resulting in minimal further error reduction in the re-interpolated maps (defined as an error reduction rate of <0.05 ms per mesh node per additional point sampled). This threshold represents a LAT interpolation error of 1% assuming maximal sampling density of 4 points/cm^2^ and typical conduction velocity of 1 ms^−^^1^. Calculation of the error reduction rate was made as shown in *Figure **2*. First, each map was re-interpolated with point sampling densities in the range 0.25–10 points/cm^2^ (step size 0.125 points/cm^2^, simulated data repetitions = 100; clinical data repetitions = 10). For each repetition, sample points were distributed randomly across the mapping domain. The mean LAT map accuracy (see above) for each density was plotted against sampling density and a power law of the form $f\left(s\right)=as^{b}+c$ was fitted to the resulting mean accuracies (where s is the sampling density and a, b, and c are fitted parameters). A power law was chosen owing to the observed distribution of the average LAT map error and to permit an analytical determination of optimal sampling density that was less sensitive to noise. Taking the first derivative of this function (i.e. $f^{'}\left(s\right)=abs^{b-1}$) gives the improvement in map accuracy per node relative to an increase in sampling density. This equation was solved for $f^{'}\left(s\right)=-0.05$ ms/node/Δdensity, in order to determine the sampling density at which minimal further improvement in map accuracy is achieved by further point sampling.

### Electroanatomic surface area calculation

Left atrial cardiac chamber models were exported from the Carto3 mapping system and imported into MATLAB 8.2 (The Mathworks Inc., Natick, MA) using custom-written software. The total surface area of the chamber was calculated as the summation of the areas of all triangles within a distance of 10 mm of an original mapping point. The threshold of 10 mm was chosen to delineate electrically inactive regions (e.g. superior/inferior caval veins, pulmonary veins) from the chamber body and is analogous to setting a similar value as the colour fill threshold of the EAM platform.

### Data analysis and statistics

Custom software was written for determining the optimal LAT sampling point density (MATLAB 8.2, The Mathworks Inc., Natick, MA). Data analysis was performed using Prism version 6.0c (GraphPad Software, San Diego, California, USA). Results are presented as mean ± SD. Student’s unpaired *t*-test was used to compare group means. Multiple comparisons were performed with the one-way analysis of variance (ANOVA). Proportions in two or more groups were compared with the Chi-square test. A significance level of *P* < 0.05 was considered statistically significant.

## Results

### Clinical left atrial tachycardias

**Figure 3:**
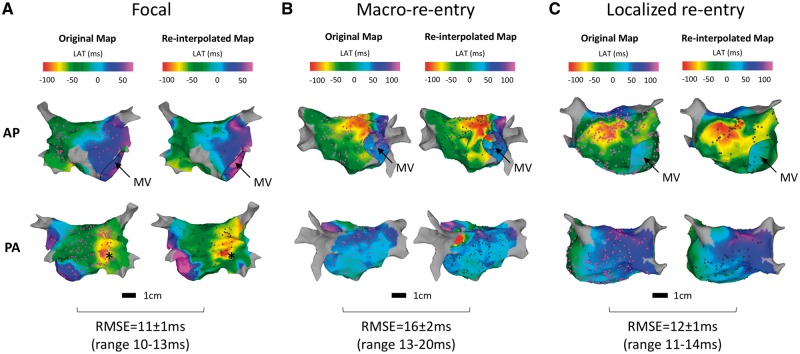
Clinical isochronal local activation time maps. Local activation time (LAT) maps for example clinical tachycardias are shown. Tachycardia mechanisms (confirmed by response to entrainment manoeuvres and to ablation) are given as headings in each column. (*A*) focal activation in low posterior LA; (*B*) perimitral macro-re-entry; (*C*) localized anterior re-entry. Anterior-posterior and posterior-anterior projections are shown (top and bottom row respectively). Left-most maps of each pair are the original isochronal maps produced at the time of the clinical procedure with purple dots representing sampled LAT point locations. Right-most maps of each pair are single examples of re-interpolated maps generated at the calculated optimal densities with black dots representing the re-sampled point locations. MV indicates the mitral valve annulus; * indicates the site of focal activation.

Local activation time maps representing 21 left atrial activation patterns were studied including focal activation (*n* = 8), localized re-entry (*n* = 5) and macro-re-entry (*n* = 8) (see *Figure **3*). Focal activation included four maps created during pacing, from the left atrial appendage (*n* = 1) and coronary sinus (*n* = 3). There were no significant differences in symptomatology, co-morbidities, or use of anti-arrhythmic drugs between the groups (*Table **1*). The mean LA surface area was 156 ± 44 cm^2^ with an original sampled point density of 5.6 ± 4.2 points/cm^2^. Optimal LAT sampling density was lower for focal tachycardias than both localised re-entry and macro-re-entry tachycardias (calculated optimal sampling densities 0.67 ± 0.17 points/cm^2^ for focal tachycardias, 1.05 ± 0.32 points/cm^2^ for macro-re-entrant tachycardias and 1.23 ± 0.26 points/cm^2^ for localized re-entry, *P* = 0.0031, *Figure **1*B). Calculated optimal sampling density was insensitive to the selected value of error reduction rate (*Table **2*). The focal tachycardia maps re-interpolated at optimal LAT sampling density (LAT error rate reduction <0.05 ms/node/point) resulted in correct localization of the foci of earliest activation (*Figure **3**A*). In the re-entrant tachycardias re-interpolation at optimal LAT sampling density resulted in the same overall map appearance, with the same macro-re-entrant circuit remaining identifiable (*Figure **3**B*). However, some variations in map appearance at the extremes of interpolation (e.g. beyond the pulmonary vein ostia) were identifiable. The localized re-entrant maps re-interpolated at optimum sampling density (*Figure **3**C*) were remarkably similar in appearance to the original maps; but some variations were evident in activation timing extrapolated into the pulmonary veins.

**Table 1:** Patient characteristics

	Focal activation	Localized re-entry	Macro re-entry	*P*
	(*n* = 8)	(*n* = 5)	(*n* = 8)	
Symptoms				
Palpitations	8 (100%)	3 (60.0%)	7 (87.5%)	0.1317
Breathlessness	0 (0.0%)	1 (20.0%)	2 (25.0%)	0.3301
Tachycardia-associated cardiomyopathy	0 (0.0%)	1 (20.0%)	1 (12.5%)	0.4581
Co-morbidities				
Atrial fibrillation	7 (87.5%)	5 (100%)	7 (87.5%)	0.7079
Hypertension	2 (25.0%)	2 (40.0%)	2 (25.0%)	0.8106
Diabetes mellitus	0 (0.0%)	1 (20.0%)	1 (12.55)	0.4581
Stroke/TIA	1 (12.5%)	0 (0.0%)	0 (0.0%)	0.4261
Vascular disease	0 (0.0%)	0 (0.0%)	0 (0.0%)	n/a
Heart failure	0 (0.0%)	0 (0.0%)	0 (0.0%)	n/a
Coronary artery disease	1 (12.5%)	0 (0.0%)	1 (12.5%)	0.7079
Valve disease	1 (12.5%)	1 (20.0%)	0 (0.0%)	0.4581
COPD	1 (12.5%)	0 (0.0%)	0 (0.0%)	0.4261
Hypo/hyperthyroidism	1 (12.5%)	1 (20.0%)	0 (0.0%)	0.4581
Echocardiography				
LVEF (%)	62.5 ± 2.7%	58 ± 11.0%	53.3 ± 10.8%	0.2361
LA diameter (cm)	4.3 ± 1.1 cm	5.0 ± 1.1 cm	4.4 ± 0.6 cm	0.1179
LA area (cm^2^)	25.6 ± 5.5 cm^2^	31.6 ± 3.8 cm^2^	24.8 ± 5.7 cm^2^	0.1321
Prior Ablation	4 (50.0%)	5 (100%)	8 (100%)	0.0180 *
Antiarrhythmic drugs				
Current AAD use	3 (37.5%)	0 (0.0%)	3 (37.5%)	0.2691
AAD	Flecainide ×3		Flecainide ×1, Sotalol ×2	

TIA, transient ischaemic attack; COPD, chronic obstructive pulmonary disease; LVEF, left ventricular ejection fraction; AAD, antiarrhythmic drug.

**Table 2:** Sensitivity of optimal sampling density to chosen error reduction rate threshold

	ERR	OSD	Sensitivity	1/Sensitivity
	(ms/node/Δdensity)	(points/cm^2^)	(∂OSD/∂ERR)	
Focal tachycardia	0.05	0.67±0.17	0.057±0.024	18
Macro-re-entry	0.05	1.05±0.32	0.052±0.022	19
Localized re-entry	0.05	1.23±0.26	0.057±0.018	18

Throughout this study an Error Reduction Rate (ERR) of 0.05 ms/node/Δdensity is taken to represent the density at which further sampling offers limited improvement in interpolated LAT map accuracy (termed optimal sampling density, OSD). The sensitivity of OSD to changes in choice of ERR are presented as the derivative of OSD with respect to ERR. The final column (1/Sensitivity) indicates the change in ERR that would result in a change in OSD of 1 point/cm^2^.

**Figure 4:**
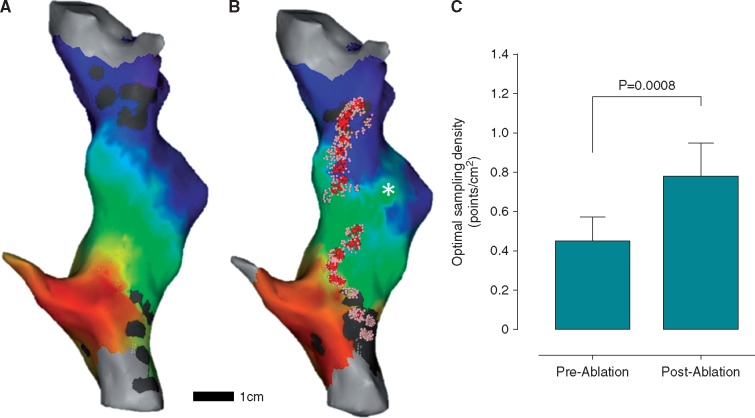
Porcine right atrial LAT maps. (*A*) Pre-ablation LAT shows uniform activation across the chamber from coronary sinus towards the right atrial appendage. (*B*) Post-ablation LAT shows a new activation detour (asterisk) via the mid-chamber gap in the ablation line. Ablation points are shown with red tags and catheter positions during ablation with pink dots using the Visitag Carto3 module. (*C*) Comparison between optimal LAT sampling density for pre- and post-ablation LAT maps.

### Porcine right atrial activation and chamber geometry complexity

Eight pre-ablation LAT maps created under proximal CS pacing were studied. In the absence of ablation, activation proceeded superiorly in a linear fashion around the tubular right atrium in all cases (*Figure **4**A*). After incomplete inter-caval linear ablation, a new activation detour was evident in all maps (*Figure **4**B*, asterisk). Increasing activation pattern complexity, created by ablation, resulted in a significant increase in the optimal LAT sampling density (0.45 ± 0.13 points/cm^2^ pre-ablation vs. 0.78 ± 0.17 points/cm^2^ post-ablation, *P* = 0.0008, *Figure **4**C*). Overall, the optimal point sampling density for the geometrically-simple porcine right atrium was significantly less than that for the geometrically-complex human left atrium (0.61 ± 0.22 points/cm^2^ vs. 1.0 ± 0.34 points/cm^2^, *P* = 0.0015).

**Figure 5:**
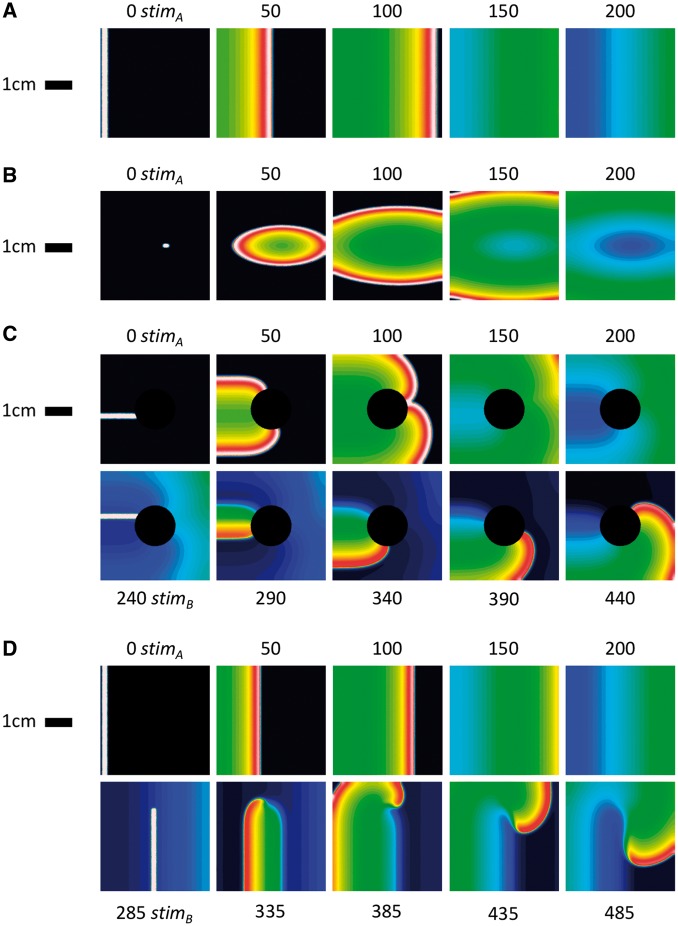
Initiation of activation patterns in the atrial monolayer*.* (*A*) Linear activation; (*B*) focal activation; (*C*) re-entrant activation; and (*D*) spiral wave activation. Stimulated regions are shown in white in the left-most column of each row.

### Simulated activation patterns

**Figure 6:**
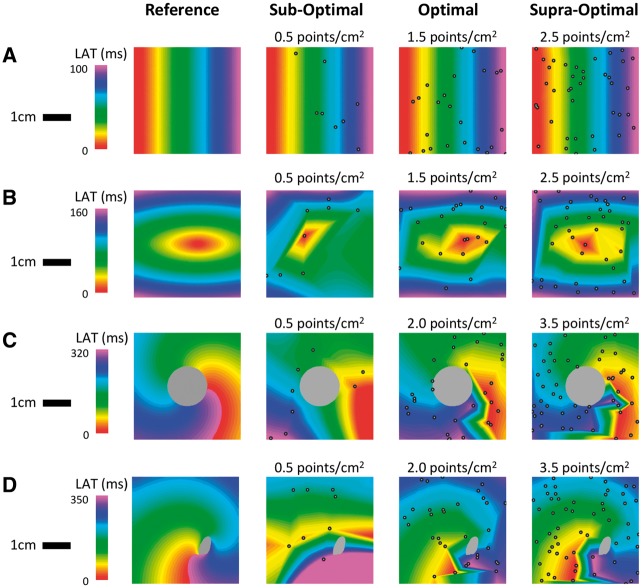
Simulated isochronal local activation time maps. (*A*) Linear activation; (*B*) focal activation; (*C*) re-entrant activation; and (*D*) spiral wave activation. Reference LAT maps are shown in the left most column. In A and B, local activation time is taken as the first activation of each node. In *C* and *D*, a window of interest (WOI) incorporating 100% of the tachycardia cycle length (TCL) was specified. Grey colour represents tissue that is never activated during the WOI—either non-conducting (*C*) or functionally refractory (*D*).

Four simulated activation patterns were studied (linear, focal, spiral-wave, and re-entrant activation). Initiation of activation is shown in *Figure **5*. Reference LAT maps were created from local activation times defined at every mesh node, resulting in an extremely high reference sampling density of ∼2500 points/cm^2^. Calculated optimal LAT sampling densities (*Figure **1**A*) for focal, re-entrant and spiral-wave activation patterns were 1.09 ± 0.14 points/cm^2^, 1.44 ± 0.49 points/cm^2^, and 1.50 ± 0.34 points/cm^2^, respectively. Linear activation in a 2-dimensional domain represents a special case where map accuracy is independent of sampling density (*Figure **6**A*).

Examples of re-interpolated isochronal LAT maps at sub-optimal, optimal, and supra-optimal LAT sampling densities for focal, re-entrant, and spiral-wave activation patterns are shown in *Figure **6**B–D*. For focal activation, sub-optimal sampling resulted in incorrect focus localization. For re-entrant activation, sub-optimal sampling density resulted in an activation pattern more consistent with a focal source. Finally, sub-optimal sampling of spiral-wave activation failed to reveal rotational activity in the resulting re-interpolated map. In each case there was limited morphological improvement in the isochronal map appearances at supra-optimal compared to optimal LAT sampling densities (*Figure **6**B–D*, third and final columns).

## Discussion

The main findings of this study are: (1) optimal LAT sampling densities can be identified for a variety of tachycardia mechanisms to maximize the diagnostic yield of interpolated local activation time maps, with a minimal sampling density of 1.0–1.5 points/cm^2^ identified for optimal interpolation of accurate LAT maps; (2) optimal LAT sampling density is dependent on activation pattern with greater sampling densities required to correctly reveal more complicated activation sequences including macro-re-entry and spiral-wave activation; and (3) optimal LAT sampling density is also dependent on chamber geometry, with increasing geometric complexity necessitating an increased optimal LAT sampling density.

The aim of tachycardia activation mapping is 2-fold: to determine the tachycardia mechanism and thereby inform an ablation strategy. The ablation target is determined by the site of tachycardia origin (focal mechanism) or the location of a critical isthmus of conduction (re-entrant mechanism). Local activation time mapping should be supplemented by consideration of the response to entrainment manoeuvres and more importantly the response to ablation when confirming the final tachycardia mechanism. Not infrequently, local activation time maps (which depend on choice of an adequate reference, window of interest and appropriate electrogram annotation) may produce conflicting information. In this context, understanding the mapping density required to achieve optimal sampling of the arrhythmia mechanism is crucial in optimising diagnostic yield of mapping prior to selection of an ablation target. Three-dimensional electro-anatomic mapping systems provide a framework for visualization of electrical activation sequences as an aid to identifying the tachycardia mechanism and corresponding ablation target. Recent technological advances have enabled significant increases in the spatial resolution achievable within reasonable timescales with both established^10^ and emerging^11^ contact mapping platforms.

Whilst high sampling density is clearly important for substrate-mapping accuracy, for example in identifying areas of low voltage or fractionated signals important to arrhythmogenesis,^12^^,^^13^ it is uncertain if high-resolution mapping results in clinical benefit during activation mapping, where the primary aim is to reliably identify the tachycardia mechanism. Indeed high-resolution activation mapping is at odds with earlier strategies aiming to minimize procedure time without impacting success by providing a logical framework for directing the acquisition of LAT points.^14^ To this end, we have shown that in systems using interpolation methods to create isochronal LAT maps from a number of sampled LAT times, a theoretical maximal sampling density is reached at which further clinically-relevant improvements in LAT map accuracy are minimal. By using both simulated data in addition to clinical data, we have demonstrated that this maximal sampling density is within the same range (∼1.0–1.5 points/cm^2^) irrespective of the resolution at which the ‘gold standard’ activation sequence is defined.

In performing this analysis, distributions of mapping points at a range of sampling densities were used to identify the global density at which further sampling yielded minimal improvement in activation map accuracy. In doing so, a random distribution of ‘re-sampling’ points was used for the following reasons. First, given the flexible nature of mapping catheters the spatial distribution of clinical mapping points is non-uniform. Secondly, the endocardial surface of the atrium is irregular resulting in non-uniform spatial distribution of mapped points even with uniform catheter electrode spacing. Finally, by repeating the randomization multiple times, and calculating error for each repeated interpolated activation map the overall calculated optimal density should be independent of variations in the spatial distribution of re-sampling points. Nevertheless, non-uniform activation mapping is likely to be important in certain clinical circumstances (e.g. focal tachycardias—see below).

The identification of atrial spiral-waves has received renewed attention recently as a strategy to identify ablation targets in atrial fibrillation.^15^ Whilst rotors have been identified in animal^16^ and computer models^17^ of atrial activation, there is some debate as to which technologies can reliably detect the occurrence of rotational activity *in vivo*.^18^^,^^19^ Clinical approaches to identifying rotors typically involve phase mapping in order to average activation over many cardiac cycles. This approach has successfully identified rotors in the left atrium using a 64-pole basket catheter.^15^ Taking the left atrial endocardial surface area to be 150 cm^2^ (as in this study) and assuming maximal electrode contact, this equates to a sampling density of 0.43 points/cm^2^. A recent analysis using the basket catheter found a mean of only 31 electrodes provided interpretable electrograms.^20^ Suboptimal contact therefore results in lower sampling densities (e.g. 56 electrodes equates to 0.37 points/cm^2^, 48 electrodes to 0.32 points/cm^2^, and 32 electrodes to 0.21 points/cm^2^). All of these densities are below the optimal LAT sampling density for spiral-wave activity identified in this study. Of note an alternative approach using dynamic voltage mapping applied to identical clinical data failed to identify sustained rotational activity in 100% (*n* = 11) of cases.^19^ This result is consistent with our finding that a significantly higher sampling density of around 1.5 points/cm^2^ should be required to optimally reproduce spiral-wave activity from known local activation time points.

The results presented here should not be interpreted to indicate that sampling densities above 1.5 points/cm^2^ are never of clinical benefit in activation mapping. One limitation of the ‘optimally’ resampled clinical maps shown in *Figure **3* is that interpolation at regions distant to the body of points (e.g. extending into the pulmonary veins or LAA) may be inaccurate. Furthermore a local density of >1.5 points/cm^2^ may well be required to correctly identify the origin of focal or localised-re-entrant tachycardias. Such variable distributions of mapping points have not been examined in this study. Rather, the results of this study support the hypothesis that once an upper global sampling density of 1.5 points/cm^2^ has been reached (equivalent to around 225 points for a typical LA with endocardial surface area of 150 cm^2^) then tachycardia mechanisms should be evident or strongly suggested, and, if so, subjected to further testing which may include more detailed activation mapping (for candidate focal or localized re-entry mechanisms) or entrainment mapping (for candidate macro-re-entry mechanisms). Furthermore, where the site of earliest activation through an incomplete linear lesion is sought, higher density mapping at the planned ablation target may be of benefit.^4^

### Limitations

Whilst this study has assessed mapping density for atrial arrhythmia contact mapping using typical electrode sizes/configurations used in clinical practice, the impact of alternative electrode size on optimal sampling density has not been examined and should be addressed in future studies. The clinical population studied in this manuscript is small and represents a select group of patients. In particular, we highlight (*Table **1*) that the cases were characterised by mild underlying structural heart disease and were predominantly cases having undergone prior ablation.

## Conclusions

In conclusion, this work demonstrates that for commonly-encountered clinical tachycardia mechanisms the majority of information discernible from LAT maps can be obtained with a limited sampling density strategy. The exact sampling density required depends on tachycardia mechanism and clinical characteristics but is in the range of 1.0–1.5 points/cm^2^. In the event where candidate mechanisms are not identifiable at this sampling density, rather than collecting further points a better approach may be to begin a new activation map perhaps with a different window of interest, reference electrogram, or LAT assignment technique.

**Conflict of interest**: none declared.

## Funding

This work was supported by the British Heart Foundation (PG/13/37/30280), an unrestricted educational grant from Biosense Webster and the National Institute for Health Research (NIHR) Biomedical Research Centre at Guy’s and St Thomas’ NHS Foundation Trust and King’s College London. The views expressed here are those of the authors and not necessarily those of the NHS, the NIHR or the Department of Health.
